# In Silico Identification of Potential Antagonists Targeting the HPV16 E2-E1 Interaction: A Step Toward Novel Therapeutics for Cervical Cancer

**DOI:** 10.3390/cimb47040288

**Published:** 2025-04-18

**Authors:** Jesús Alonso Gandara-Mireles, Verónica Loera Castañeda, Julio Cesar Grijalva Ávila, Ignacio Villanueva Fierro, Cynthia Mora Muñoz, Hugo Payan Gándara, Guadalupe Antonio Loera Castañeda, Leslie Patrón Romero, Horacio Almanza Reyes

**Affiliations:** 1 CIIDIR-Durango Unit, National Polytechnic Institute, Genomics Academy, Durango 34220, DG, Mexico; veronica.loera@gmail.com (V.L.C.); jcgrijalva69@gmail.com (J.C.G.Á.); ignacio.villanueva.fierro@gmail.com (I.V.F.); 2 Precipharm, Personalized and Precision Medicine Laboratory, Durango 64753, DG, Mexico; quimico.antonio.loera@gmail.com; 3 Epidemiology Service, State Center of Cancerology, CECAN Durango, Durango 34000, DG, Mexico; cynthia.mora@ujed.mx (C.M.M.); drpayangandara17@gmail.com (H.P.G.); 4 Faculty of Medicine and Psychology, Autonomous University of Baja California, Tijuana 22390, BC, Mexico; patron.leslie@uabc.edu.mx (L.P.R.); almanzareyes@hotmail.com (H.A.R.)

**Keywords:** human papillomavirus, cervical cancer, antiviral, antagonist molecules

## Abstract

*Human papillomavirus* (HPV) infection is the most prevalent sexually transmitted disease, and a primary cause of persistent infection leading to cervical cancer (CC). CC remains one of the most common malignancies among women worldwide, with approximately 660,000 new cases and 350,000 deaths annually. In Mexico, this cancer accounts for 13.9% of female deaths. Currently, no antiviral treatment exists for HPV infection. Available therapies for dysplasia and CC focus on the destruction or surgical removal of infected tissue using cytotoxic agents. While the prophylactic HPV vaccine effectively prevents new infections, it does not benefit the millions already infected, underscoring the urgent need for novel therapeutic strategies. This study aimed to identify potential antagonists for the interaction between the *HPV16* E2 and E1 proteins through in silico screening. A virtual screening was performed targeting the TAD of the *HPV16* E2 protein (PDB ID: 1DTO) using the Maybridge HitFinder™ small molecule library. Six molecules with the best binding energies were identified: 11419, 11829, 10756, 10708, 10632, and 10726. Among these, molecules 10756, 10708, 10632, and 10726 demonstrated promising potential as antagonists, interacting with Tyr19 and/or Glu39 residues. These findings highlight potent therapeutic candidates against *HPV*-related diseases.

## 1. Introduction

*Human papillomavirus* (HPV) infection is the most common sexually transmitted disease, affecting both men and women. Persistent HPV infection is the primary causative agent of cervical cancer (CC) [1,2]. Globally, CC is among the most prevalent cancer types in women, with approximately 660,000 new cases and 350,000 deaths reported in 2022 [3]. Notably, 80% of these deaths occur in developing countries. In Mexico, CC accounts for 13.9% of female mortality [4]. Annually, approximately 33,000 new cancer cases are detected in anatomical sites commonly associated with HPV, including the penis, vagina, anus, oral cavity, and throat. In 2010, the Mexican Ministry of Health reported 13,000 cases and 6000 deaths, with an incidence rate of 14.7 cases per 100,000 women in the state of Durango [5]. According to Mexico’s National Institute of Statistics, Geography, and Informatics (INEGI), cancer was responsible for 38,000 female deaths in 2010, representing approximately 13% of all female mortality in the country. Between 1998 and 2010, cancer-related mortality increased by 35% (2.91% annually), with the mortality rates rising from 59.3 to 66.5 per 100,000. Breast cancer (13.3%), CC (10.4%), liver cancer (7.3%), and stomach cancer (6.8%) remain the leading causes of cancer-related deaths among Mexican women [5].

Although antiviral therapeutic vaccines targeting HPV have been developed, their efficacy remains limited to specific clinical scenarios, and they are not yet universally effective or widely accessible [6]. Current therapies for dysplasia and CC still rely predominantly on the destruction or surgical removal of infected tissue using cytotoxic agents or surgical interventions [7]. Additionally, while the prophylactic HPV vaccine effectively prevents new infections, it does not benefit the millions of men and women already infected. This highlights the ongoing need for innovative therapies, drugs, and procedures capable of addressing HPV infection and its complications more effectively, with the aim of achieving greater precision and accessibility in treatment options.

A promising approach to addressing HPV is the development of protein inhibitors targeting critical factors involved in its pathogenesis [8]. Identifying the lead molecules that specifically interact with HPV oncoproteins, human tumor suppressor genes implicated in viral interactions, or both is crucial for achieving highly precise and effective treatments for HPV-induced CC. The *HPV16* E2 protein plays a crucial role in regulating viral replication and transcription by interacting with the viral helicase E1 [9]. This interaction is essential for the recruitment of E1 to the origin of replication, facilitating viral genome replication. The transactivation domain (TAD) of E2 is particularly important in this process, as it mediates the protein–protein interactions necessary for viral propagation. The disruption of the E2-E1 interaction has been proposed as a potential antiviral strategy [10], as it could hinder viral replication and reduce the persistence of high-risk HPV infections. Given that persistent *HPV16* infection is a major etiological factor in cervical cancer development, targeting this interaction presents a promising avenue for therapeutic intervention. In silico strategies have emerged as powerful tools for identifying potential inhibitors of protein–protein interactions. These methods allow for the structural modeling of viral complexes and the prediction of binding affinities for candidate molecules, accelerating the early phases of drug discovery [11,12]. In this study, we applied advanced molecular docking techniques, supported by structural validation using AlphaFold and PyMOL, to explore potential antagonists of the *HPV16* E2-E1 interaction. The objective of our study was to identify inhibitory molecules capable of antagonizing the interaction between the *HPV16* E2 and E1 proteins, a critical step in the viral replication process. To achieve this, we employed in silico screening methods, focusing on the TAD of the *HPV16* E2 protein as the therapeutic target. Using computational approaches and advanced molecular docking techniques, this work aims to contribute significantly to the development of innovative therapeutic strategies for the treatment of HPV-associated CC.

## 2. Materials and Methods

### 2.1. Selection of Binding Pocket

To identify potential inhibitory molecules targeting the TAD of HPV16 E2, we employed a multi-step computational methodology combining molecular docking simulations, ligand preparation, and sequence alignment analyses. This approach began with the acquisition and preparation of the target protein structure for virtual screening, followed by the selection of a chemically diverse library of small molecules. The selection of the binding pocket was guided by its essential role in the E2-E1 interaction, a key process in HPV replication. Using the Molecular Operating Environment (MOE) software, we identified a biologically relevant binding site based on structural evidence and a residue conservation analysis. The selected molecules were optimized for the docking simulations by generating stable conformers and assigning partial charges. Thousands of docking poses were generated and evaluated based on their binding energies to the TAD. Additionally, sequence alignments were performed to explore conserved residues across different HPV types, providing insights into the structural conservation of the TAD and reinforcing its potential as a therapeutic target.

#### 2.1.1. Previous Evidence on the Relevance of the TAD in the E2-E1 Interaction

The TAD of the E2 protein is crucial in regulating the replication and transcription of the HPV genome. Previous studies have shown that this domain interacts with the viral helicase E1 [13,14], facilitating its recruitment to the origin of replication. Disrupting this interaction represents a viable strategy to inhibit viral replication and prevent the progression of persistent *HPV16* infection, the most prevalent high-risk type in cervical cancer.

#### 2.1.2. Identification of Binding Site Using MOE

We used the MOE software version 2022.02 (Chemical Computing Group Inc., Montreal, QC, Canada) to analyze the surface of the E2 protein and detect regions with hydrophobic and hydrophilic characteristics suitable for ligand interaction. Through the Site Finder algorithm, we identified potentially functional cavities, prioritizing those containing key residues previously implicated in the protein–protein interactions relevant to viral replication.

#### 2.1.3. The Structural Conservation of the TAD Across Different HPV Types

To assess the biological relevance of the selected binding site, we performed a sequence alignment analysis of the TAD across multiple high-risk HPV types (*HPV16, HPV18, HPV31, HPV33*, and *HPV45*). We found that approximately 60% of the residues within the selected cavity were highly conserved, including Tyr19 and Glu39, which have been reported as essential for interaction with the E1 protein. This conservation suggests that the ligands identified in our study could have a functional impact on multiple virus variants.

### 2.2. Target Acquisition and Preparation

The three-dimensional structure of the HPV16 E2 TAD was obtained from the Protein Data Bank (PDB, www.rcsb.org accessed on (13 December 2024)). The structure was then prepared for virtual screening by removing the water molecules, adding hydrogen atoms, and assigning partial charges using the Gasteiger method. This method estimates partial charges based on the partial updates of electronegative orbitals. The molecular recognition site was identified using the Site Finder application included in the MOE software version 2022.02 (Chemical Computing Group Inc., Montreal, QC, Canada) suite [15].

### 2.3. Ligand Selection and Preparation

The small molecule library used was the Maybridge Hit Finder™ collection, sourced from Thermo Fisher Scientific Inc., Waltham, MA, USA [16], which consists of 14,400 molecules representing a diverse chemical space. All molecules in the library comply with Lipinski’s rule of five, qualifying them as potential drug candidates.

The ligands were prepared using Open Babel (version 3.1.1) for format conversion and structural optimization. Additionally, AutoDockTools (version 1.5.6) was used to prepare the input files for docking, including the addition of polar hydrogens, assignment of partial charges using the Kollman method, and definition of rotatable bonds.

### 2.4. Conformer Search

Stable conformational structures were identified for the 14,400 molecules in the Hit Finder collection, excluding conformers with energies exceeding 3 kcal/mol. These conformers were then prepared for molecular docking simulations by adding hydrogen atoms and assigning partial charges using the Gasteiger method, making them suitable for the molecular recognition processes.

### 2.5. Molecular Recognition Simulations

The molecular docking simulations were performed using AutoDock Vina (version 1.1.2). The docking protocol was set up with a grid size of 40 × 40 × 40 Å, centered on the binding cavity identified in the protein. The global search algorithm was employed with an exhaustiveness parameter of 8, ensuring a thorough conformational search.

For each ligand, 80,000 poses were generated, and the binding energies were calculated using the London equation:

(1) $$∆G=C+Eflex+\sum \left(fHB\cdot CHB\right)+\sum \left(fM\cdot CM\right)+\sum Di$$

where

*C*: Average gain/loss in rotational and translational entropy.
*E*(flex): Energy associated with loss of ligand flexibility.
*f*(HB): Geometric imperfections in hydrogen bonds (value = 0.1).
*C*(HB): Energy of ideal hydrogen bond.
*f*(M): Geometric imperfections in metal–ligand interactions (value = 0.1).
*C*(M): Energy of ideal metal–ligand bond.
*Di*: Desolvation energy of atom *i*.

This comprehensive approach allowed for the identification and evaluation of ligands with strong binding potentials to the HPV16 E2 TAD, contributing to the discovery of novel therapeutic candidates.

### 2.6. Docking Validation

To ensure the reliability of the docking protocol, a validation process was performed by comparing the docking results with the co-crystallized structures available in the literature for similar proteins; verifying that the ligand conformations in the top-ranked docking poses aligned with those in the crystallographic structures; conducting re-docking experiments to confirm the reproducibility of the docking predictions; and comparing the binding energy values with the experimental data reported in previous studies to evaluate the accuracy of the docking calculations.

### 2.7. Preprocessing and Ligand Optimization

To enhance the docking accuracy, the ligands were optimized before docking using:

• Energy minimization with MMFF94 force field in Avogadro (version 1.2.0).
• Protonation state correction at physiological pH using Epik server (Schrödinger).

### 2.8. Selection Criteria for Best Binding Poses

The binding energy scores obtained from AutoDock Vina were evaluated for steric compatibility with the identified binding cavity and interaction compatibility with the key residues previously identified as crucial for the HPV16 E2-E1 interaction.

### 2.9. Search for TAD Sequences of HPV E2

The amino acid sequences of the TAD of HPV E2 from types 16, 18, 33, 35, and 45 were retrieved from the National Center for Biotechnology Information (NCBI) database (www.ncbi.nlm.nih.gov accessed on 3 April 2025). After obtaining the sequences, pairwise alignments (HPV16 against each of the other sequences) were performed to determine the identity and homology percentages using the GGSEARCH/GLSEARCH program (http://fasta.bioch.virginia.edu/fasta_www2/fasta_www.cgi?rm=select&pgm=gnw accessed on 3 April 2025). Additionally, multiple sequence alignments were conducted to identify conserved residues within the TAD of the E2 protein for HPV types 18, 33, 35, and 45 using the T-Coffee program (http://www.ebi.ac.uk/Tools/msa/tcoffee accessed on 1 April 2025).

### 2.10. Structural Modeling and Protein–Ligand Interaction Analysis

We conducted a multi-level structural validation to assess the selection of the binding pocket and the orientation of the identified compounds, following a three-step approach for the generation of an HPV16 E1-E2 complex model.

We used AlphaFold [17] to simulate the E1-E2 replication complex based on FASTA sequences (GenBank: AYV61476.1, AYV61477.1) obtained from the NCBI. This approach allowed us to visualize the initial conformation of the complex and confirm the accessibility of the TAD in the E2 protein.

#### 2.10.1. TAD Marking in E2

To highlight the location of the TAD, the selection and coloring tool in PyMOL [18] was used, where the key residues identified in the structural analysis were marked in red. This visualization allowed for a more precise delineation of the binding pocket and facilitated the evaluation of ligand accessibility to the active site.

#### 2.10.2. Ligand Selection, Evaluation, and Molecular Docking

As previously mentioned, six compounds of interest (ID: 11419, 11829, 10756, 10708, 10632, and 10726) were selected based on physicochemical criteria and their compliance with Lipinski’s rule, indicative of potential bioactivity. The interaction prediction of these compounds with the TAD cavity was performed using AutoDock Vina, defining a search grid centered on the key residues identified in the structural analysis.

The docking poses obtained were analyzed in PyMOL, focusing on the stability of the ligands within the binding site. The protein–ligand interactions were identified by measuring the distances between the compounds and critical residues within the TAD cavity, considering hydrophobic interactions and hydrogen bonding.

## 3. Results

### 3.1. Target Preparation

**Figure 1:**
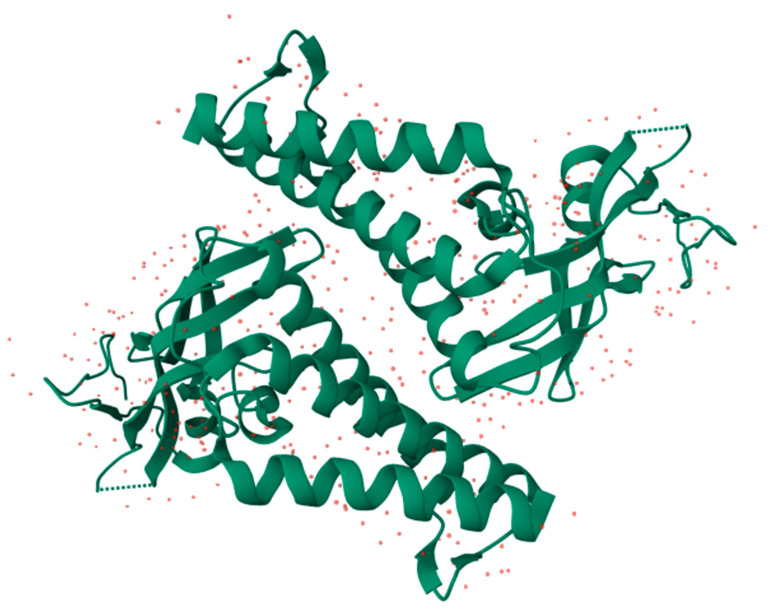
The 3D structure of the transactivation domain (TAD), PDB-ID: 1DTO [19]. The figure highlights the overall domain fold and surface topology used to identify the potential binding zones for small molecule docking.

Figure 1 shows the structure of the transactivation domain (TAD) of the HPV16 E2 protein, obtained from the Protein Data Bank (PDB-ID: 1DTO) [19]. A computational surface analysis of the protein was performed using the Site Finder application in the MOE software version 2022.02 (Chemical Computing Group Inc., Montreal, QC, Canada). This tool utilizes an algorithm to identify concave regions on the protein surface and assesses whether these cavities have an appropriate composition of hydrophobic and hydrophilic atoms.

The selected molecular recognition cavity included the following residues: Ile15, Tyr19, Asp28, His29, Ile30, Tyr32, Trp33, Met36, Glu39, Ala63, Val64, Ser65, Asn67, Lys68, Ala69, Gln71, Ala72, Leu79, Thr93, Leu94, Gln95, Val97, Ser98, Leu99, Glu100, and Val101.

### 3.2. Conformer Search

The conformational search for the most stable structures among the 14,400 molecules resulted in a total of 63,267 conformers.

### 3.3. Molecular Recognition

Molecular recognition simulations were performed on the TAD of HPV16 using a compound library and the MOE software version 2022.02 (Chemical Computing Group Inc., Montreal, QC, Canada). From these simulations, the 31 best molecules were initially selected based on their binding energy scores (Table 1).

### 3.4. Structural Analysis

**Figure 2:**
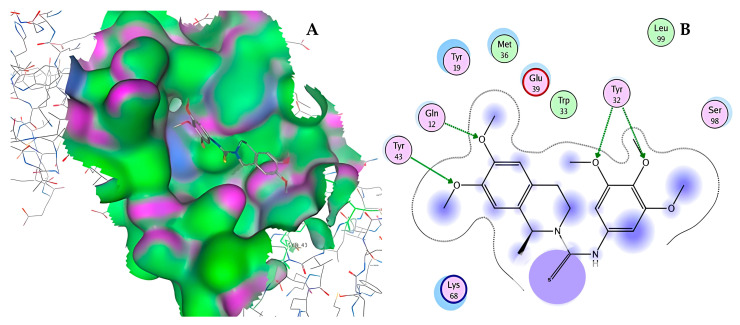
The interactions between compound 11419 and the TAD binding cavity of the HPV16 E2 protein. (**A**) The docking pose of compound 11419 within the TAD, showing its position at the central region of the binding pocket. (**B**) A two-dimensional interaction map illustrating hydrogen bonds between compound 11419 and the residues Tyr32, Tyr43, and Gln12. The residues are color-coded based on their chemical properties: the red circles indicate negatively charged residues, such as Glu39; the blue circles denote positively charged residues, like Lys68; the green circles represent nonpolar or hydrophobic residues, including Trp33 and Leu99; and the purple or pink circles highlight polar uncharged residues, such as Tyr, Gln, and Ser. This pattern of hydrogen bonding and polar contacts suggests the stable interaction of the compound within the TAD cavity, supporting its potential role in disrupting the E2-E1 protein interface.

**Figure 3:**
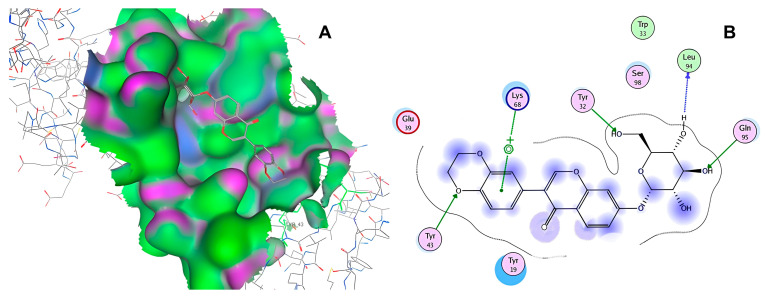
The interactions between compound 11829 and the TAD binding cavity of the HPV16 E2 protein. (**A**) The docking pose of compound 11829 within the transactivation domain (TAD), showing its spatial orientation and positioning inside the central region of the binding pocket. (**B**) A two-dimensional interaction map illustrating the hydrogen bonds formed between compound 11829 and the key residues Tyr32, Tyr43, Leu94, Lys 68, and Gln95. These residues are in regions functionally relevant to the interaction with the E1 helicase. The chemical nature of each residue is highlighted through color coding: negatively charged residues, such as Glu39, are shown with red circles; positively charged residues like Lys68 appear in blue; hydrophobic residues, such as Leu94, appear in green; and polar uncharged residues, including Tyr, Ser, and Gln, are marked in pink or purple.

**Figure 4:**
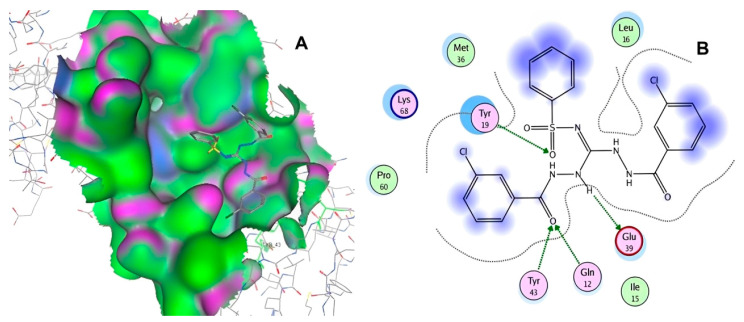
Interactions between compound 10756 and TAD binding cavity of HPV16 E2 protein. (**A**) Docking pose of compound 10756 within transactivation domain (TAD), showing its orientation in binding pocket surface, in close contact with central region of cavity. (**B**) Two-dimensional interaction map depicting hydrogen bonds formed between compound 10756 and residues Tyr19, Tyr43, Gln12, and Glu39. These residues are functionally relevant in E2-E1 interface and contribute to stabilization of ligand. Residue types are color-coded: red-circled residues are negatively charged, blue-circled residues (Lys68) are positively charged, green-circled residues (Met36, Leu16, Ile15) are nonpolar/hydrophobic, and pink or purple circles denote polar uncharged residues.

**Figure 5:**
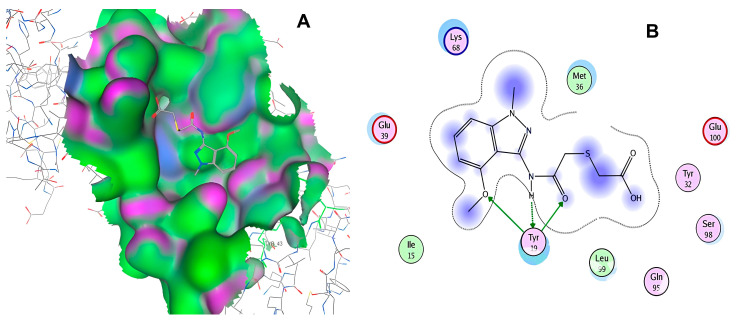
The interactions between the binding cavity residues and molecule 10708. (**A**) The ocking of molecule 10708 in the TAD of HPV16 E2. (**B**) A 2D map of the interactions between the compound and the amino acid residues in the TAD of HPV16 E2. The interactions between compound 10708 and the TAD binding cavity of the HPV16 E2 protein. (**A**) The docking pose of compound 10708 within the transactivation domain (TAD), revealing its orientation inside the central pocket region of the binding surface. (**B**) A two-dimensional interaction map showing the hydrogen bonds formed between the ligand and the residue Tyr19, a key component of the E2 interaction interface. The surrounding residues are color-coded by chemical type: those in red circles are negatively charged; the blue circles are positively charged; thee green circles are hydrophobic; and the purple circles are polar uncharged.

**Figure 6:**
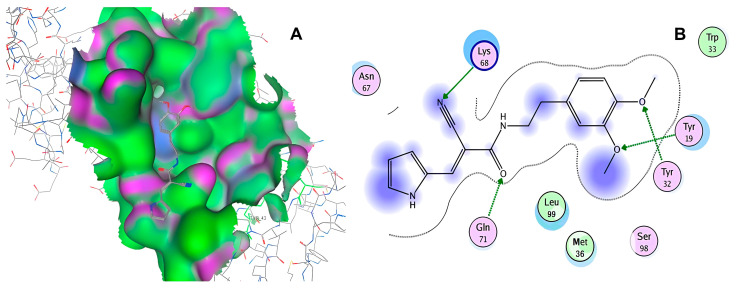
Interactions between compound 10632 and TAD binding cavity of HPV16 E2 protein. (**A**) Docking pose of compound 10632 within transactivation domain (TAD), showing its location inside binding pocket and its proximity to structurally relevant residues. (**B**) Two-dimensional interaction map illustrating hydrogen bonds between compound 10632 and residues Gln71, Lys68, Tyr32, and Tyr19. These interactions suggest strong polar anchoring of ligand within cavity. Residues are color-coded by their chemical natures: positively charged residues are shown in blue circles; hydrophobic residues appear in green; and polar uncharged residues are marked in purple or pink.

**Figure 7:**
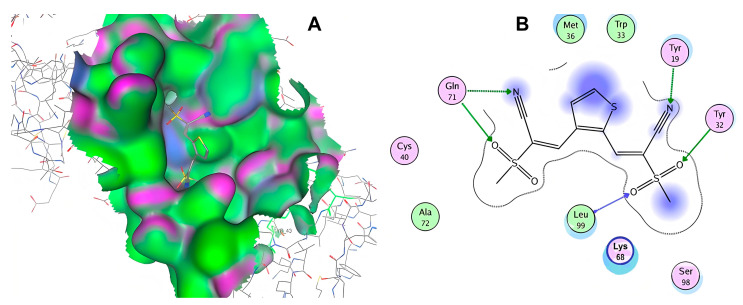
The interactions between compound 10726 and the TAD binding cavity of the HPV16 E2 protein. (**A**) The docking pose of compound 10726 within the transactivation domain (TAD), showing its position nestled in the central region of the cavity surface. (**B**) A two-dimensional interaction map illustrating hydrogen bonds between the compound and the residues Gln71, Leu99, Tyr19, and Tyr32. These residues play important roles in protein–protein recognition, particularly in the E2-E1 interaction. The residues are color-coded according to their chemical natures: polar uncharged residues are shown in pink; positively charged residues are marked in blue; and hydrophobic residues appear in green.

After selecting the molecules with the highest binding energies, a structural analysis was conducted to examine their binding modes and interactions within the TAD binding cavity of the HPV16 E2 protein. Six molecules were identified, among which compounds 11419 and 11829 demonstrated significant binding potentials (−14.3274 and −14.0887 kcal/mol, respectively) within the selected TAD cavity. Both molecules interacted with the amino acid residues Tyr 32 and Tyr 43, as illustrated in Figure 2 and Figure 3. Additionally, molecules 10756, 10708, 10632, and 10726 displayed reasonable binding potentials (−13.1464, −12.6922, −12.5200, and −12.3050 kcal/mol, respectively) in the same TAD cavity. These compounds formed hydrogen bonds with the residues Tyr 43, Tyr 32, Tyr 19, and Leu 99, as shown in Figure 4, Figure 5, Figure 6 and Figure 7.

**Figure 8:**
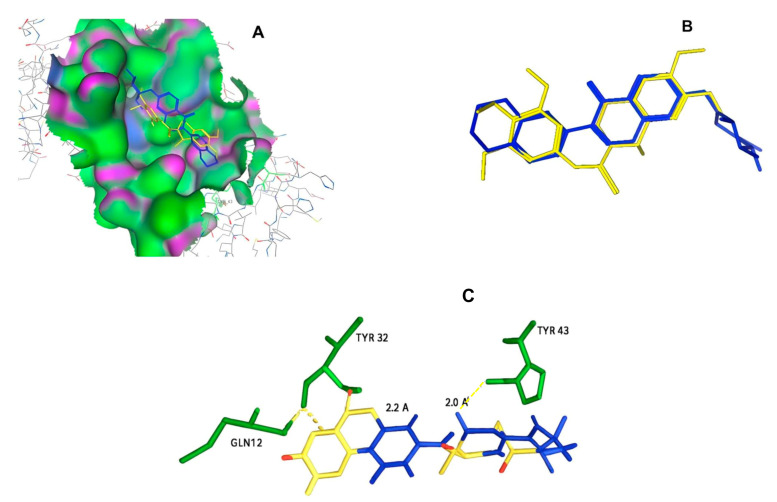
The interactions between the binding cavity residues and molecules 11419 and 11829. (**A**) The binding modes of molecules 11419 (yellow) and 11829 (blue). (**B**) The flexible alignments of compounds 11419 (yellow) and 11829 (blue). (**C**) The Tyr32, Tyr43, and Gln12 residues that interact with the ligands; similarities are observed in the docking positions of molecules 11419 (yellow) and 11829 (blue) with the residues (green) in the binding site. Details of the specific hydrogen bonding interactions (yellow dotted line) between the compounds and the key residues Tyr32, Tyr43, and Gln12. The bond distances (2.0 Å and 2.2 Å) reinforce the stability of the binding within the TAD cavity.

**Figure 9:**
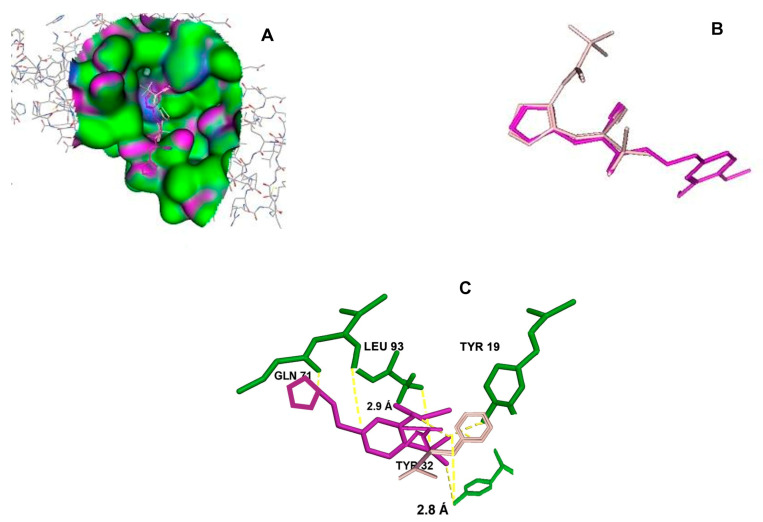
Interactions between binding cavity residues and molecules 10632 and 10726. (**A**) Binding modes of molecules 10632 (magenta) and 10726 (pink). (**B**) Flexible alignments of molecules 10632 (magenta) and 10726 (pink). (**C**) Molecules 10632 (magenta) and 10726 (pink) with residues (green) in binding site. Detailed representation of binding geometry and hydrogen bonds (yellow) formed by both ligands with key residues, including bond distances (2.8 Å and 2.9 Å). This view provides clearer spatial context for stabilizing interactions observed in binding pocket.

The structural analysis revealed that compounds 11419 and 11829, in addition to sharing common interactions, exhibited similar docking configurations within the binding cavity (Figure 8A). A flexible alignment confirmed the excellent overlap of the molecules (Figure 8B). Figure 8C illustrates their docking positions and shared interactions within the binding site, providing a detailed view of the specific hydrogen bonding interactions between both the ligands and the key residues Tyr32, Tyr43, and Gln12. The measured distances (2.0 Å and 2.2 Å) support the presence of strong and stable hydrogen bonds within the TAD binding pocket, reinforcing the proposed inhibitory mechanism. This visualization addresses the spatial arrangement of the residues and the interaction geometry more clearly and directly. Compounds 10632 and 10726 were docked within the central region of the HPV16 E2 TAD binding cavity, exhibiting distinct yet overlapping binding modes (Figure 9A). The flexible alignments of both ligands (Figure 9B) revealed a partial structural similarity, suggesting that specific subregions of the molecules may adopt comparable conformations during binding. A detailed interaction analysis (Figure 9C) showed that both compounds established hydrogen bonds with key residues such as Tyr32, Gln71, and Leu93. The measured bond distances, 2.8 Å for Tyr32 and 2.9 Å for Gln71, indicate strong stabilizing interactions that may contribute to the retention of these ligands within the pocket. These shared contacts reinforce the potential functional relevance of these residues in ligand recognition and suggest a conserved binding mechanism.

**Figure 10:**
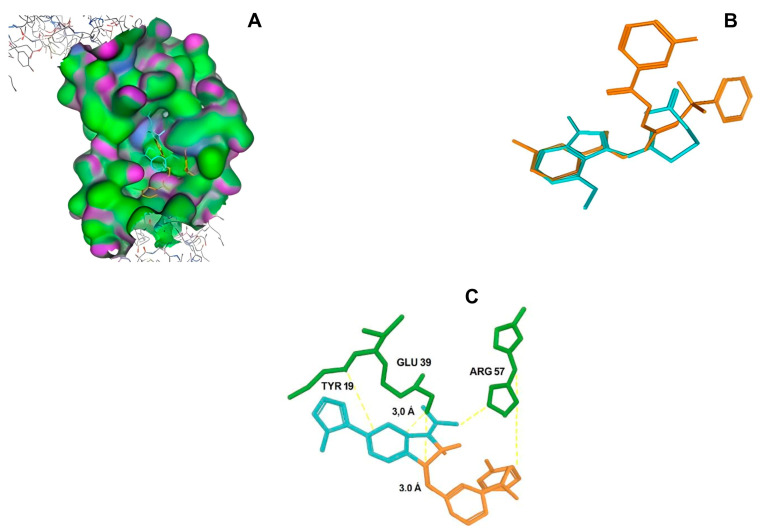
Interactions between binding cavity residues and molecules 10708 and 10756. (**A**) Binding modes of molecules 10708 (blue) and 10756 (orange). (**B**) Flexible alignments of molecules 10708 (blue) and 10756 (orange). (**C**) Interactions (dotted lines) of molecules 10708 (blue) and 10756 (orange) with residues (green) in binding site. Detailed close-up of molecular interactions showing hydrogen bonds (yellow) between each compound and key residues. Distances between atoms (2.8 Å and 3.0 Å) are shown, highlighting interaction strength and stabilizing potential of both ligands within active site.

Figure 10 depicts the interactions between the binding cavity residues and molecules 10708 and 10756. Figure 10A shows the binding modes of 10708 (blue) and 10756 (orange) at the center of the binding cavity, indicating that both occupy similar positions. Figure 10B presents the flexible alignments of these molecules, highlighting the partially overlapping regions that suggest relevant structural similarities. Figure 10C illustrates the interactions (dotted lines) formed by the molecules with the binding site residues (green), emphasizing the common interactions that may play a crucial role in the biological activity within this region, and provides a detailed visualization of the hydrogen bond network formed by compounds 10708 and 10756 with the critical residues within the TAD cavity. Specifically, 10708 formed hydrogen bonds with Tyr19 and Arg57, while 10756 showed interactions with Glu39 and Tyr19. The measured bond distances (2.8 Å and 3.0 Å) support the strength of these interactions.

Table 2 provides the Lipinski parameter values for the selected molecules, indicating that they meet the established criteria to be considered drug-like candidates.

### 3.5. Sequence Alignment of HPV E2 TAD

**Figure 11:**
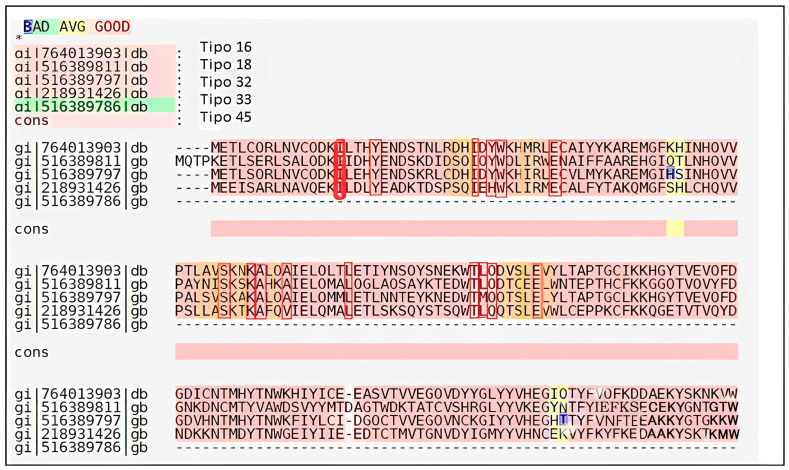
Multiple sequence alignment of TAD of E2 protein from HPV types 16, 18, 31, 33, and 45. Residues highlighted in red represent highly conserved amino acids across all five HPV types. Residues highlighted in yellow are partially conserved or show conservative substitutions. Residues highlighted in orange correspond to those identified in our docking and interaction studies (e.g., Tyr19, Glu39, Arg57) as key participants in ligand binding. This color scheme highlights both general sequence conservation and specific functional relevance of residues within binding pocket.

Once the amino acid sequences of the TAD from HPV subtypes 16, 18, 31, 33, and 45 were identified, pairwise alignments were performed between HPV16 E2 and each of the other subtypes. The results of the multiple sequence alignment are presented in Figure 11. The highlighted residues in the alignments (Figure 11) correspond not only to the conserved positions, but also to those involved in protein–ligand interactions, as identified through our docking analysis.

### 3.6. Analysis of HPV16 E2 TAD and Ligand Interactions

**Figure 12:**
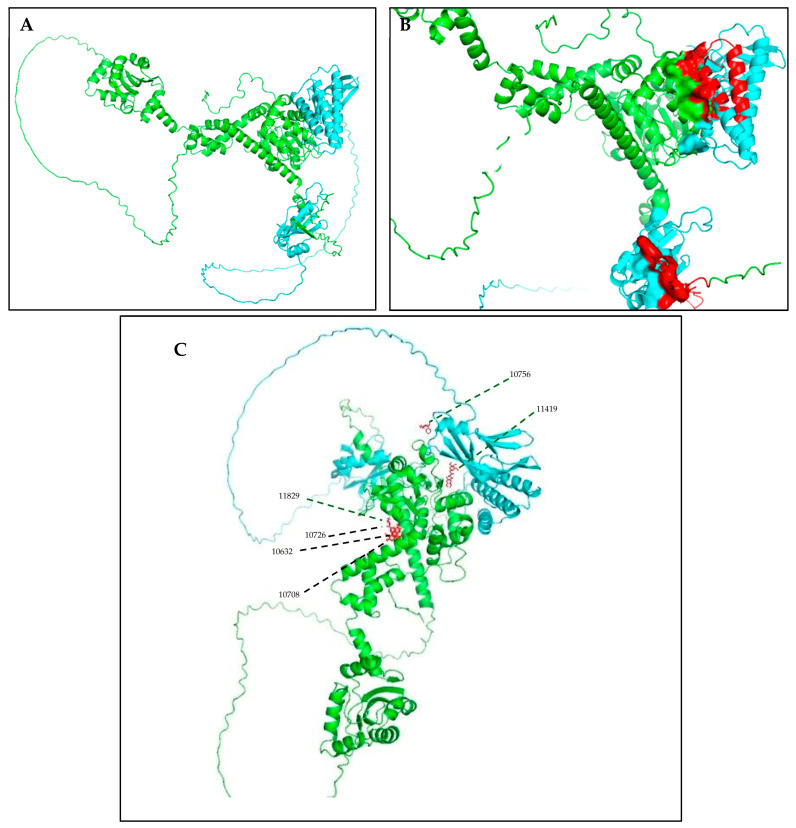
The structural modeling of the HPV16 E1-E2 replication complex and analysis of the protein–ligand interactions in the E2 TAD. (**A**) A three-dimensional model of the HPV16 E1-E2 complex generated using AlphaFold, based on FASTA sequences retrieved from the NCBI database. The E1 protein is shown in green, and E2 in blue. The model illustrates the spatial configuration and domain interactions essential for viral replication (prediction confidence scores: pLDDT > 90, pTM = 0.62, ipTM = 0.59). (**B**) A close-up of the transactivation domain (TAD) of E2, highlighting the predicted binding pocket residues in red. These residues were identified as critical for compound interaction through virtual screening and structural analysis. A visualization was performed using PyMOL to assess the pocket accessibility and ligand compatibility. (**C**) Molecular docking simulations showing the spatial positioning of the six selected compounds within the E2 binding cavity. The compounds are labeled (10756, 11419, 10708, 11829, 10632, and 10726) and shown in stick representation. Clustered binding near the center of the cavity was observed for compounds 11829, 10632, 10726, and 10708, while compounds 11419 and 10756 were oriented more specifically within the TAD region. Compound 11419 displayed the widest binding distribution and the strongest predicted interactions, with hydrogen bond distances ranging from 2.5 to 3.5 Å.

The structural analysis of the E1-E2 complex allowed us to identify the cavity within the transactivation domain (TAD) of E2 (Figure 12A–C), characterized by the presence of key residues involved in small molecule interactions. The molecular docking analysis in PyMOL revealed that four of the six selected compounds (IDs: 11829, 10632, 10726, and 10708) clustered in the central region of the model, while compounds 11419 and 10756 were specifically positioned within the TAD cavity. Among these, compound 11419 exhibited the highest stability within the binding site, with an average binding energy of −7.8 kcal/mol. This compound established key interactions with the residues Glu39, Arg57, and Tyr85, forming hydrogen bonds with Glu39 (2.8 Å) and Arg57 (3.2 Å). Additionally, hydrophobic interactions were identified with Leu42 and Val91, contributing to the stability of the complex.

The protein–ligand distance analysis confirmed that the orientation of compound 11419 within the TAD cavity favors a stable interaction, optimally positioning the ligand to functionally block E2 activity.

## 4. Discussion

The interaction between the E2 protein and the E1 helicase of HPV is crucial for initiating viral DNA replication [20]. Several studies have highlighted the essential role of the residue Glu39 in both its interaction with the viral E1 protein and the replication process [21,22,23,24,25]. In this study, six molecules were selected based on their binding potentials and key interactions within the binding cavity. Four of these molecules formed hydrogen bonds with the residues Tyr32 and Tyr19. Tyrosine, commonly found in the E2 proteins of high-risk HPV types, plays a critical structural and functional role in stabilizing specific interactions within the binding cavity. Among the four molecules interacting with tyrosine, two exhibited the highest binding potentials, while the other two demonstrated reasonable binding potentials.

These findings are consistent with previous reports emphasizing the functional role of tyrosine residues in E2-mediated protein interactions. In our multiple sequence alignment including high-risk HPV types 16, 18, 32, 33, and 45 we observed that critical residues such as Tyr19, Tyr32, Glu39, and Arg57 were highly conserved. This conservation supports the hypothesis that the selected ligands may be effective across multiple high-risk HPV types by targeting structurally similar features within the E2 TAD binding cavity [26]. Although low-risk HPV types were not included in our alignment, the focus on clinically relevant high-risk types aligns with the primary objective of identifying therapeutic candidates against the forms of HPV most strongly associated with cervical cancer. In our results, four molecules (10756, 10708, 10632, and 10726) interacted with Tyr19, with 10756 forming hydrogen bonds with Glu39. This interaction may potentially block Glu39’s interaction with the E1 protein, thereby preventing the initiation of viral DNA replication.

Abbate et al. (2004) [27], in their crystallographic study of the HPV18 E1-E2 complex, identified a critical salt bridge between the residue Glu43 (E2) and Arg454 (E1), whose disruption through mutation abolishes the interaction between these two proteins. Although our analysis was based on the HPV16 E2 protein (PDB ID: 1DTO), it is important to note that Glu39 in HPV16—structurally and functionally conserved aligns with Glu43 in HPV18, as confirmed by our sequence alignments. In our results, compound 10756 formed hydrogen bonds with Glu39 (Figure 12C), and compound 11419 also interacted with Glu39 and Arg57, suggesting that these molecules could interfere with the critical salt bridge between E2 and E1, thereby preventing the formation of the replication complex. This finding reinforces the therapeutic potential of the identified molecules, as they not only anchor within the TAD cavity, but could also directly disrupt one of the most essential interactions for viral replication.

The pairwise alignments showed a high percentage of homology between the analyzed HPV types (18, 31, 33, and 45). The multiple sequence alignment results revealed that approximately 60% of the amino acid residues forming the selected cavity in the TAD of E2 were conserved, including Tyr19 and Gln39. Thus, it is reasonable to hypothesize that any of the four molecules interacting with these residues could block interactions not only in HPV16, but also in other high-risk HPV types.

Previous research on the inhibition of the HPV E1–E2 interaction has demonstrated that small molecules could effectively disrupt DNA replication in low-risk HPV types by targeting the E2 transactivation domain (TAD). Notably, Davidson et al. (2004) [28] described a series of indandione-containing compounds that inhibited the DNA replication in low-risk HPV types by reversibly binding to the E2 transactivation domain (TAD) and preventing its interaction with E1. Using photoaffinity labeling and mass spectrometry, they identified a precise binding region near the residue Met101 of the E2 protein. Their Compound I displayed structural features, such as a planar aromatic core and functional groups compatible with hydrogen bonding, that bear a substantial resemblance to compound 10708, identified in our study. This structural similarity is particularly interesting, as both compounds are predicted or demonstrated to bind in overlapping regions of the E2 pocket. Additionally, White et al. (2011) [29] showed high-affinity binding to the E1 binding interface on E2. Structure–activity relationship studies yielded compounds with nanomolar potency against the E1-E2 interaction and micromolar activity in cell-based replication assays. Despite the significant progress made, these efforts were eventually discontinued. While no specific public explanation was provided for halting the program, the subsequent literature has suggested that limitations related to in vivo potency, pharmacokinetics, or selectivity may have hindered further development. Our study revisits this same protein protein interaction target using updated computational tools, including AlphaFold-based structural prediction and refined virtual screening, and identifies novel scaffolds such as compound 10708 that share key binding features with the previous lead compounds [30]. This continuity highlights the therapeutic relevance of the E1–E2 interface and reinforces its viability as a druggable site for future antiviral strategies.

One major strength of this study is the selection and analysis of molecules with high binding potentials within the TAD cavity of the E2 protein, enabling the identification of key interactions with conserved residues like Tyr19 and Gln39. Additionally, using multiple alignments to compare high-risk HPV types strengthens the hypothesis that these molecules may inhibit the E1/E2 interaction across multiple HPV types. In our work, we conducted an analysis using AlphaFold and PyMOL to validate the structure of the complex and the accessibility of the selected compounds to bind within the TAD binding cavity. We found that compounds 11829 and 10756 were specifically positioned within the TAD cavity, with compound 11419 standing out for exhibiting the highest stability in the binding site at an average binding energy of −7.8 kcal/mol. This compound established key interactions with the residues Glu39, Arg57, and Tyr85, forming hydrogen bonds with Glu39 (2.8 Å) and Arg57 (3.2 Å). These findings position 11419 as a promising candidate for further studies.

Previous studies, such as that by Soumia et al. [31], have employed similar approaches, proposing in silico strategies to identify small molecules inhibiting E6-E6AP interactions. In contrast, our study focuses on an in silico screening targeting the most prevalent HPV type associated with cervical cancer. However, a potential limitation is the lack of experimental validation of these inhibitory interactions in vivo, which would provide more direct evidence of their effectiveness in blocking E1/E2 interactions and preventing viral replication [32]. Further studies, including in vitro assays [33,34] or experiments using animal models [35], are needed to confirm the biological relevance of the observed interactions and evaluate the clinical potential of the selected molecules. To experimentally validate the predicted inhibitory interactions, several in vitro approaches could be employed. Among these, traditional assays such as Southern blot, qPCR, and luciferase reporter systems are widely used to assess viral genome replication [36,37]. More recently, infection models using quasiviruses combined with qPCR have emerged as highly informative systems for evaluating the impacts of antiviral compounds on the early stages of the viral life cycle [38]. Given that the compounds identified in this study are predicted to interfere with the E2-E1 interaction, an essential step for replication origin recruitment quasivirus-based assays or replication-competent reporter systems may provide the most suitable platforms to test their inhibitory potential under controlled cellular conditions.

In addition to the experimental validation mentioned in previous studies, our selection of ligands was based on structural stability criteria and compatibility with the key residues in the binding pocket, further reinforcing the validity of the molecular docking approach. Moreover, the applicability of this method has been supported by numerous studies in identifying inhibitors of protein–protein interactions, particularly in cases where full crystallographic structures are not available. As part of our future research plans, we will not only incorporate molecular dynamics simulations to assess the temporal stability of the formed complexes, but will also explore advanced computational modeling techniques, such as hybrid QM/MM methods, to improve the accuracy of our predictions and prioritize the most promising compounds for experimental evaluation [39,40,41,42,43,44,45,46].

## 5. Conclusions

This study identifies promising small molecules with significant binding potentials within the TAD binding cavity of the HPV16 E2 protein, highlighting their potential as inhibitors of viral DNA replication. Compounds 11419 and 11829 demonstrated the strongest binding affinities (−14.3274 and −14.0887 kcal/mol, respectively) and exhibited consistent interactions with conserved residues, such as Tyr32 and Tyr43. Four additional molecules (10756, 10708, 10632, and 10726) displayed reasonable binding potentials (−13.1464 to −12.3050 kcal/mol) and interacted with the residues critical for stabilizing the protein–protein interactions essential for viral replication, including Tyr19 and Glu39. The structural analyses revealed that the binding configurations of 11419 and 11829 overlapped significantly, suggesting common mechanisms of action. Similarly, 10708 and 10756 displayed shared interactions and partial overlap within the binding cavity, further underscoring the key structural features that may underpin biological activity. The validation analyses of the complex structure and the accessibility of the selected compounds to bind within the TAD binding cavity showed that compounds 11829 and 10756 were specifically positioned within the TAD cavity. Notably, compound 11419 exhibited the highest stability within the binding site, positioning it as a promising candidate for further studies.

Our findings are consistent with previous structural studies, such as that of Abbate et al. (2004) [27], which identified a key salt bridge between Glu43 (E2) and Arg454 (E1) in HPV18. In our model, compound 10756 formed bonds with Glu39—the functionally homologous residue in HPV16—suggesting that these compounds could interfere with such electrostatic interactions, which are critical for the formation of the E1-E2 complex. This potential disruption of the salt bridge represents an additional mechanism by which the selected ligands may inhibit viral replication.

The sequence alignment analyses demonstrated a high degree of conservation in the TADs across high-risk HPV types, particularly at the residues Tyr19 and Glu39, supporting the hypothesis that these molecules could inhibit the E1/E2 interactions across multiple HPV subtypes. Overall, this study provides compelling in silico evidence supporting the potential of these compounds as inhibitors of HPV replication. However, the lack of experimental validation highlights the need for further in vitro and in vivo studies to confirm their efficacy and assess their clinical applicability. These findings represent an important step toward the development of targeted therapeutics aimed at disrupting HPV replication and addressing HPV-associated malignancies.

## Figures and Tables

**Table 1:** A description of the 31 selected molecules based on their binding energies.

ID	Chemical Structure	Name	Binding Energy (Kcal/mol)
11419	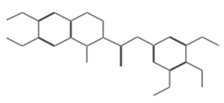	N2(3,4,5-trimethoxyphenyl)-6,7-dimethoxy-1-methyl-1,2,3.	−14.3274
11829	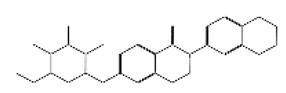	Beta-D-glucopyranose	−14.0887
10756	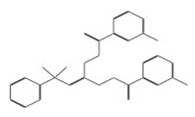	N1-di[2-(3-chlorobenzoyl)hydrazino]methylene benzene-1-sulfonamide	−13.1464
11277	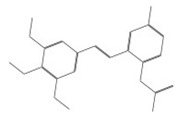	N1-{4-methyl-2-[(3,4,5-trimethoxybenzylidene)amino]phenylacetamide	−13.0737
11667	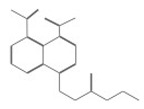	4-[(2-ethoxy-2-oxoethyl)thio]naphthalene-1,8-dicarboxylic acid	−12.9282
11048	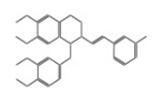	1-(3,4-dimethoxybenzyl)-2-[(2-fluorophenyl)diazenyl]-6,7-dimethoxy-1,2,3,4-tetrahydroisoquinoline	−12.9047
11044	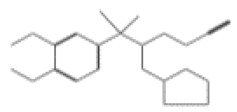	N-(2-cyanoethyl)-3,4-dimethoxy-N-(tetrahydrofuran-2-ylmethyl)benzenesulfonamide	−12.8421
10635	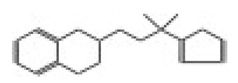	N-(2,3-dihydro-1,4-benzodioxin-2-ylmethyl)-2-thiophenesulfonamide	−12.8407
11773	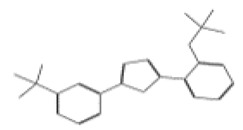	2-[2-(trifluoromethoxy)phenyl]-5-[3-(trifluoromethyl)phenyl]-1,3,4-oxadiazole	−12.8036
10708	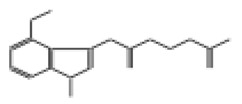	2-({2-[(4-methoxy-1-methyl-1H-indazol-3-yl)amino]-2-oxoethyl}sulfinyl)acetic acid	−12.6922
10167	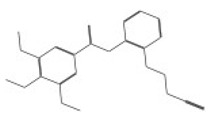	N1-{2-[(2-cyanoethyl)thio]phenyl}-3,4,5-trimethoxybenzamide	−12.6907
11091	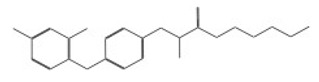	2-ethoxyethyl 2-{4-[(3,5-dichloro-2-pyridyl)oxy]phenoxy}propanoate	−12.6658
11744	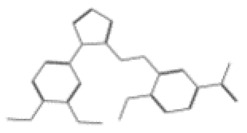	1-[3-({[1-(3,4-dimethoxyphenyl)-1H-1,2,3,4-tetrazol-5-yl]thio}methyl)-4-methoxyphenyl]ethanone	−12.6091
11080	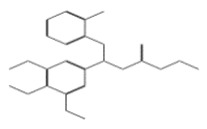	Ethyl 3-[(2-aminophenyl)thio]-3-(3,4,5-trimethoxyphenyl)propanoate	−12.5635
10632	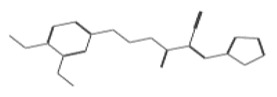	N1-(3,4-dimethoxyphenyl)-2-cyano-3-(1H-pyrrol-2-yl)acrylamide	−12.5200
10006	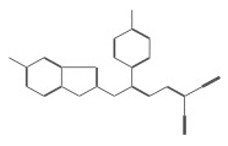	4-benzyl-2-({4-[5-(trifluoromethyl)-2-pyridyl]piperazin-1-yl}methyl)morpholine difumarate	−12.4922
11821	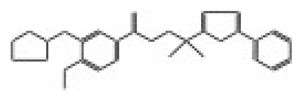	N’-[3-(cyclopentyloxy)-4-methoxybenzoyl]-5-(2-pyridyl)-2-thiophenesulfonohydrazide	−12.4093
11987	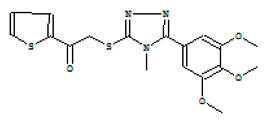	4H-1,2,4-triazol-3-yl}sulfinyl}-1-(2-thienyl)-1-ethanone	−12.3364
10726	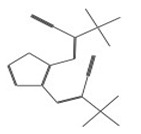	3-{2-[2-cyano-2-(methylsulfonyl)vinyl]-3-thienyl}-2-(methylsulfonyl)acrylonitrile	−12.3050
10007	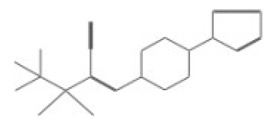	2-(tert-butylsulfonyl)-3-[4-(1H-pyrrol-1-yl)piperidino]acrylonitrile	−12.2512
11385	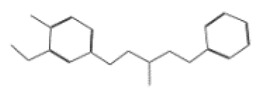	4-[3-(benzylamino)butyl]-2-methoxyphenol	−12.1643
10531	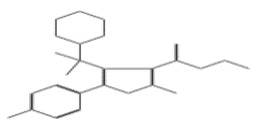	Ethyl 2-methyl-5-(4-methylphenyl)-4-(morpholinosulfonyl)-3-furoate	−12.1102
11275	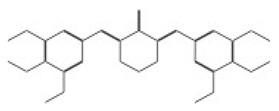	3,5-di(3,4,5-trimethoxybenzylidene)tetrahydro-2H-pyran-4-one	−12.0896
11821	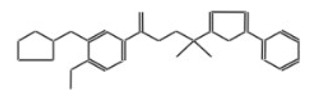	3-[2-(2-pyridyl)ethyl]-2-thioxoimidazolidin-4-one	−12.0887
10745	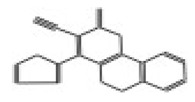	4-(2-furyl)-2-oxo-1,5-dihydro-2H-chromeno[4,3-b]pyridine-3-carbonitrile	−12.0696
11642	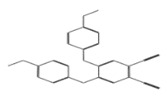	4,5-di(4-methoxyphenoxy)pentanedinitrile	−12.0528
11765	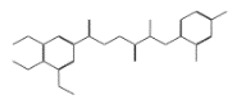	N’1-[2-(2,4-dichlorophenoxy)propanol]-3,4,5-trimethoxybenzene-1-carbohydrazide	−12.0513
11688	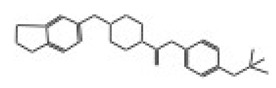	4-(1,3-benzodioxol-5-ylmethyl)-N-[4-(trifluoromethoxy)phenyl]tetrahydro-1(2H)-pyrazinecarboxamide	−12.0467
11277	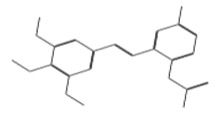	N1-{4-methyl-2-[(3,4,5-trimethoxybenzylidene)amino]phenyl}acetamide	−12.0318
10711	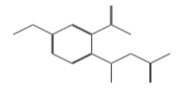	2-[(carboxymethyl)(methyl)amino]-5-methoxybenzoic acid	−12.0258
10026	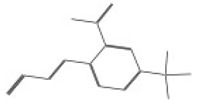	3-[2-nitro-4-(trifluoromethyl)phenyl]acrylonitrile	−12.0242

**Table 2:** The Lipinski parameter values for the selected molecules.

No.	ID	Molecular Weight g/mol	Hydrogen Donors	Hydrogen Acceptors	log P
1	11419	432.5380	1	7	2.8000
2	11829	458.4170	4	10	0.4600
3	10756	506.3680	4	9	3.7000
4	10708	309.3450	2	7	1.2700
5	10632	325.3660	2	6	1.0700
6	10726	342.4190	0	6	0.0000

## Data Availability

The data presented in this study are available upon request from the corresponding authors due to their proprietary restrictions and ongoing use for research and development.
